# Transcription Factor IAA27 Positively Regulates P Uptake through Promoted Adventitious Root Development in Apple Plants

**DOI:** 10.3390/ijms232214029

**Published:** 2022-11-14

**Authors:** Shuo Zhao, Xuewen Zhao, Xuefeng Xu, Zhenhai Han, Changpeng Qiu

**Affiliations:** 1 College of Horticulture, China Agricultural University, Beijing 100193, China; 2 Key Laboratory of Stress Physiology and Molecular Biology for Fruit Trees in Beijing Municipality, China Agricultural University, Beijing 100193, China

**Keywords:** adventitious root, low phosphorus stress, *Malus domestica*, *MdIAA27*

## Abstract

Phosphate (P) deficiency severely limits the growth and production of plants. Adventitious root development plays an essential role in responding to low phosphorus stress for apple plants. However, the molecular mechanisms regulating adventitious root growth and development in response to low phosphorus stress have remained elusive. In this study, a mutation (C-T) in the coding region of the apple *AUXIN/INDOLE-3-ACETIC ACID 27 (IAA27)* gene was identified. *MdIAA27T*-overexpressing transgenic apple improved the tolerance to phosphorus deficiency, which grew longer and denser adventitious roots and presented higher phosphorous content than the control plants under low phosphorus conditions, while the overexpression of *MdIAA27C* displayed the opposite trend. Moreover, the heterologous overexpression of *MdIAA27* in tobacco yielded the same results, supporting the aforementioned findings. In vitro and in vivo assays showed that MdIAA27 directly interacted with AUXIN RESPONSE FACTOR (ARF8), ARF26 and ARF27, which regulated *Small Auxin-Up RNA 76 (MdSAUR76)* and *lateral organ boundaries domain 16 (MdLBD16)* transcription. The mutation in IAA27 resulted in altered interaction modes, which in turn promoted the release of positive ARFs to upregulate *SAUR76* and *LBD16* expression in low phosphorus conditions. Altogether, our studies provide insights into how the allelic variation of *IAA27* affects adventitious root development in response to low phosphorus stress.

## 1. Introduction

Phosphate (P) is a vital macronutrient that plays an indispensable role in plant growth, development and major metabolic processes. Nevertheless, a large proportion of P interacts with cations (Fe^3+^, Mg^2+^, Ca^2+^ and Al^3+^), resulting in unavailability for plant uptake. The P content in soil is less than 10 μM [1,2], which can lead to P starvation and limit plant growth and survival. In agricultural production, phosphorus deficiency leads to an imbalance of soil nutrients, which in turn limits the yield potential of multiple crops. Wheat yield is frequently constrained by low phosphorus stress, such as in Australia and other tropical and subtropical regions [3,4,5]. It was also found that phosphorus deficiency could reduce the yield of cotton [6] and soybean crops [7]. Production losses caused by phosphorus are widely reported, especially in some populous countries in the world, such as India, China and the USA [8]. Under phosphorus deficiency conditions, plants have adapted in multiple pathways, such as changing the root growth and architecture [9,10,11], regulating lipid remodeling [12] and enhancing the excretion of organic acids and RNases [13]. A particularly sophisticated strategy is for plants to modify their root structure under phosphorus deficiency. Previous reports had shown that phosphorus stress has a significant influence on modulating the root system architecture (RSA), including the suppression of primary root growth [14], increments in the length and number of lateral roots (LRs) or adventitious roots (ARs) [15], production of a number of cluster roots [16] and enhancement of the density of root hairs [17]. In various plants, roots that derive from non-root tissues, such as stems and leaves, are called adventitious roots. Adventitious roots play a critical role in plant survival under biotic and abiotic stresses [18]. In field conditions, the soil nutrient uptake ability of trees with primary and adventitious roots was higher than that with only initial roots [19]. It has been reported that the initiation and elongation of AR is promoted in many plant species under low phosphorus stress, such as *Solanum lycopersicum* [20], *Populus ussuriensis* [21], *Oryza sativa* [22,23] and *Hordeum vulgare* [24]. The effect of low phosphorus stress on rice root growth is found to stimulate adventitious root growth, which increases the available root surface area and contributes to nutrient uptake [25]. Most of the previous studies on adventitious root development have centered on herbaceous plants, with little research on woody plants.

Auxin signaling is closely linked with the remodeling of the root architecture under P deficiency. The AUXIN/INDOLE-3-ACETIC ACID (Aux/IAA) proteins, as responsive factors, play vital roles in auxin signaling. Aux/IAA proteins have been revealed to bind with AUXIN RESPONSE FACTOR (ARFs) and suppress the expression of auxin-response genes in the absence of auxin. In contrast, under high auxin levels, Aux/IAA proteins interact with SCF^TIR1/AFB^, which labels and triggers their ubiquitination and degradation via the 26S proteasome and subsequently releases ARF to activate the expression of auxin downstream responsive genes [26]. Recently, studies on various plants uncovered that the Aux/IAA transcription factors (TFs) are indispensable for the auxin signaling mechanism to regulate root development. For example, in maize, *ZmIAA10* played a critical role in the regulation of seminal and lateral root initiation [27]. In Populus, *puIAA4*, downstream of *bZIP53*, inhibited adventitious root development [28]. Additionally, Aux/IAA TFs are also involved in biotic and abiotic stress responses. *AtIAA5*, *AtIAA6* and *AtIAA19* positively regulated the drought tolerance in *Arabidopsis* [29]. In rice, overexpression of *OsIAA20* positively increased the content of proline to improve drought and salt tolerance [30]. However, few studies have clarified the role of Aux/IAA in regulating adventitious root growth and development in response to P stress.

Apple (*Malus domestica*), which belongs to the Rosaceae family, occupies an important position in terms of global cultivation area and yield. Previous evidence has demonstrated that transcription factors play a pivotal role in the regulation of P-deficient responses in apple. In a previous study, *MdMYB2* positively mediated the phosphate stress response by regulating P starvation-induced (PSI) genes [31]. *MdPHR1* improved the phosphorus deficiency of apple by regulating the expression of *MdPAP10* [32]. However, the regulatory mechanism of apple Aux/IAA transcription factors in response to phosphorus stress has not been reported. Hence, it is greatly important to explore and reveal the molecular mechanism of the *Aux/IAA* genes in adventitious root development in apple rootstocks for the breeding of seedlings with more developed roots and increased resistance to phosphorus stress. In our previous bulked segregant sequence (BSA-seq) analysis (NCBI accession number: PRJNA810276, https://www.ncbi.nlm.nih.gov/bioproject/PRJNA810276 accessed on 10 November 2022) of 1002 rootstock progenies crossed from tolerant to phosphorus deficiency apple rootstocks ‘Baleng Crab’ (*Malus robusta* Rehd.; ‘BC’) and sensitive to phosphorus deficiency apple rootstocks ‘M9’ (*Malus pumila* Mill.), an auxin-responsive *Aux/IAA* gene, *MdIAA27*, was identified.

In this study, *MdIAA27* was isolated and investigated in apple ‘Baleng Crab’ (*Malus robusta* Rehd.; ‘BC’) and ‘M9’ (*Malus pumila* Mill.) plants. Gene expression profiles demonstrated that *MdIAA27* could be induced by low phosphorus treatments. Furthermore, the stable transformation experiment revealed that two linked single-nucleotide polymorphisms (SNPs) in the CDS of *MdIAA27* accounted for differences in the length and number of adventitious roots in apple and tobacco, which influenced the phosphorus uptake by roots and thus affected the low phosphorus tolerance. Moreover, IAA27 interacted with ARF8, ARF26 and ARF27, However, under low phosphorus conditions, IAA27 was degraded and thereafter released ARF26 and ARF27, which further upregulated the expression of *Small Auxin-Up RNA 76 (SAUR76)* and *lateral organ boundaries domain 16 (LBD16)* to improve the length and number of adventitious roots. In the present study, the allele mutations in IAA27 altered their interactions with ARFs and thereafter influenced adventitious root development in response to low phosphorus stress. This provided basic insights into the molecular mechanism of apple root length and root number responses to low phosphorus stress and offered valuable information for the breeding of phosphorus-tolerant apple rootstocks. Throughout the study, it was found that allele variations significantly altered the function of the IAA27 protein. Previous reports have shown that Aux/IAA was regulated by multiple transcription factors; however, whether allele variation in IAA27 would alter its interaction with other proteins is still unclear. Thus, further investigation remains to verify whether the allele variation in IAA27 affects the binding to other proteins, such as TIR1 and MYB.

## 2. Results

### 2.1. An IAA27 Mutant Allele Was Associated with Apple Rootstock Rooting in Low P Conditions

**Figure 1:**
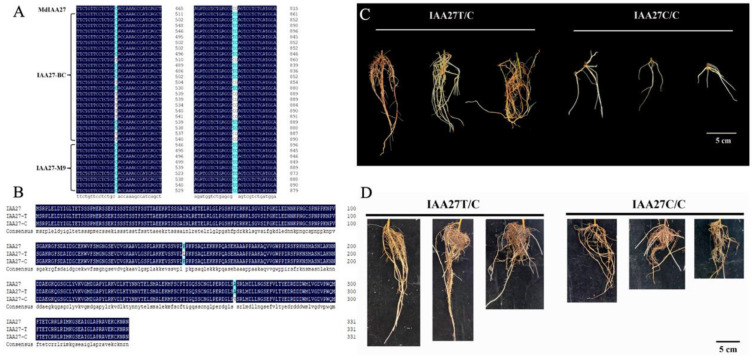
Identification of IAA27 related to root development in the parents ‘BC’ and ‘M9’ and their progenies. (**A**) Variation in the *IAA27* CDS sequence of the parents ‘BC’ and ‘M9’. (**B**) Amino acid sequence analysis of IAA27 transcription factors. (**C**) Phenotypes of adventitious roots from six progenies crossed from ‘BC’ and ‘M9’. The progenies were harvested for adventitious root trait analysis at 30 days after cutting. Scale bars: 5 cm. (**D**) After low phosphorus treatment for 30 days, adventitious roots of progenies were photographed. Scale bars: 5 cm.

Aux/IAA TF plays vital roles in plant growth and development and is also involved in responses to abiotic stresses. Based on the results of previous BSA analyses, the *MdIAA27* (MD17G1189100) gene was potentially related to the regulation of the root length and number in response to phosphorus stress. To investigate the molecular mechanisms of adventitious root development in low phosphorus conditions, the *IAA27* gene sequences from the parents ‘BC’ and ‘M9’ were obtained. This revealed a C-T SNP (Figure 1A), resulting in a non-synonymous mutation (Pro to Thr) of the protein sequence (Figure 1B), in a nuclear localization signal (NLS) contributing to degradation [33,34]. Another non-synonymous SNP TC/CG (Figure 1A) caused an amino acid substitution (Val to Ala) (Figure 1B). There was a linkage relationship between the two variants. Adventitious root development in the progenies of ‘BC’ and ‘M9’ displayed a clear pattern: T/C > C/C. We determined that the progenies with longer and denser adventitious roots were homozygous for the T nucleotide (termed the *IAA27T/C* allele); however, the progenies with less adventitious roots were heterozygous for the *IAA27C/C* allele (named the *IAA27C/C* allele) (Figure 1C). Moreover, the longer adventitious roots and higher root number were associated with increased tolerance to low phosphorus stress. After low phosphorus treatment, the adventitious root lengths and root number of *IAA27T/C* progenies were significantly higher than those of *IAA27C/C* progenies (Figure 1D).

**Figure 2:**
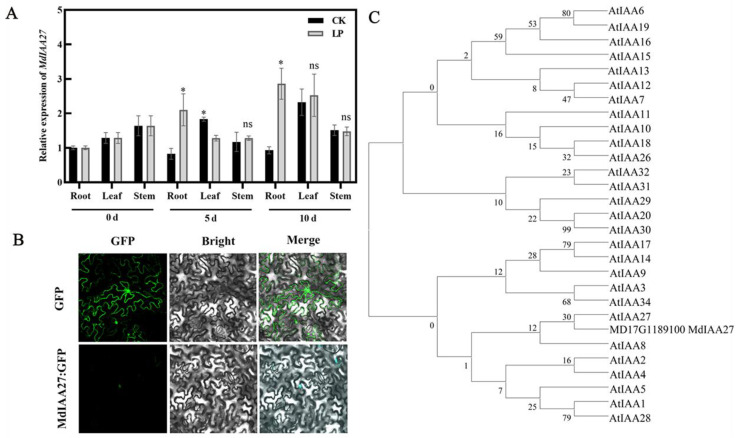
Expression characteristics of MdIAA27. (**A**) The qRT-PCR analysis of *MdIAA27* expression in root, leaf and stem under low phosphorus conditions. * indicates statistically significant differences at *p* ≤ 0.05. ns indicates no significant difference between each group. Error bars indicate standard deviation (s.d.) from three biological replicates. (**B**) Subcellular localization of MdIAA27 in tobacco leaf cells. Scale bars: 10 μm. (**C**) Phylogenetic analysis of MdIAA27 with Aux/IAA proteins from Arabidopsis.

To investigate the expression pattern of *MdIAA27*, the expression levels of *MdIAA27* in various tissues of ‘M9’ under phosphorus deficiency conditions were examined by qRT-PCR. The results showed that *MdIAA27* was expressed predominantly in the adventitious roots, and it had a similar expression trend in the leaf and stem, indicating that *MdIAA27* is mainly involved in the root response to low phosphorus stress (Figure 2A). The full-length coding sequence lacking the stop codon of *MdIAA27* was fused with green fluorescence protein (GFP) and transiently expressed under control of the 35S promoter in tobacco to verify the subcellular location of MdIAA27. The construct was introduced into tobacco leaf cells and the subcellular GFP signal was observed. The result indicated that the MdIAA27-GFP protein was localized in the nucleus (Figure 2B), showing that MdIAA27, similar to other Aux/IAA paralogs, such as SlIAA9 and GmIAA27 [35,36], is localized in the nuclei to affect the auxin response pathways. Phylogenetic analyses of MdIAA27 with the *Aux*/*IAA* gene family from *A. thaliana* were performed. The phylogenetic tree determined that MdIAA27 is closely related to AtIAA27 (Figure 2C).

### 2.2. Heterologous Expression of MdIAA27 Influenced Tolerance to Phosphate Stress in Transgenic Tobacco

**Figure 3:**
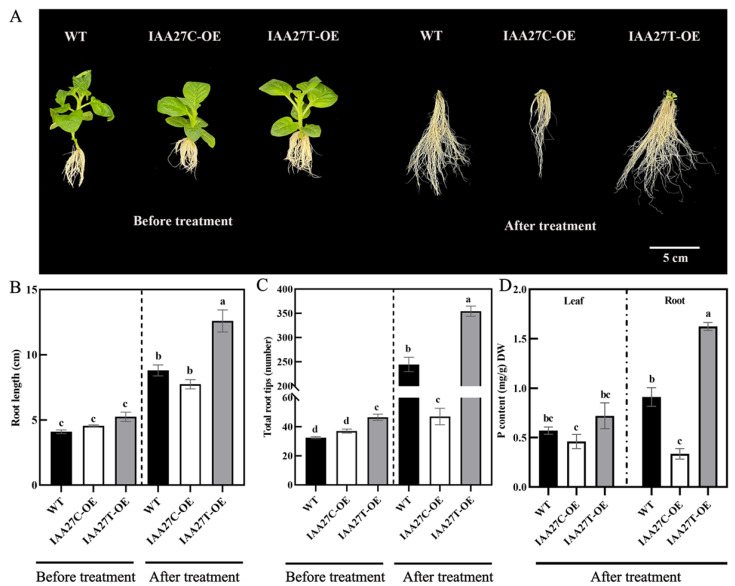
Ectopic expression of *MdIAA27* influenced tolerance to phosphate stress in transgenic tobacco. (**A**) The phenotypes of *MdIAA27* transgenic and WT tobacco in the presence of auxin (0.5 mol/L) for 15 days on MS solid medium. (**B**,**C**) Statistical analysis of total root tips and root lengths. (**D**) Phosphorus content of transgenic and WT lines after treatment with LP (10 μM KH_2_PO_4_) for 10 days. Different letters represent significant differences (*p* < 0.05). Error bars show standard deviation (s.d.) from three biological replicates.

To further verify the function of *MdIAA27*, it was overexpressed in the tobacco heterologous system. The overexpression lines (*MdIAA27T*-OE and *MdIAA27C*-OE) were generated and selected by qRT-PCR and DNA sequence analysis (Supplemental Figure S1A–C). As shown in Figure 3A, all overexpression lines exhibited slightly longer root lengths and denser root numbers than WT under phosphorus-sufficient conditions. However, *MdIAA27T*-overexpressing lines developed longer and more roots than the WT, but the root development in *MdIAA27C*-overexpressing lines was suppressed compared with the WT under phosphorus stress treatment conditions (Figure 3A–C). Consistent with the observed root phenotype, P content in the leaves and roots of *MdIAA27T* transgenic plants was also higher than that of *MdIAA27C* transgenic lines after low phosphorus treatment (Figure 3D). These results demonstrated that *MdIAA27* overexpression in tobacco influenced low phosphorus tolerance.

### 2.3. An IAA27 Mutant Allele Affected Adventitious Root Development and Changed P Deficiency Tolerance in Apple Rootstocks

**Figure 4:**
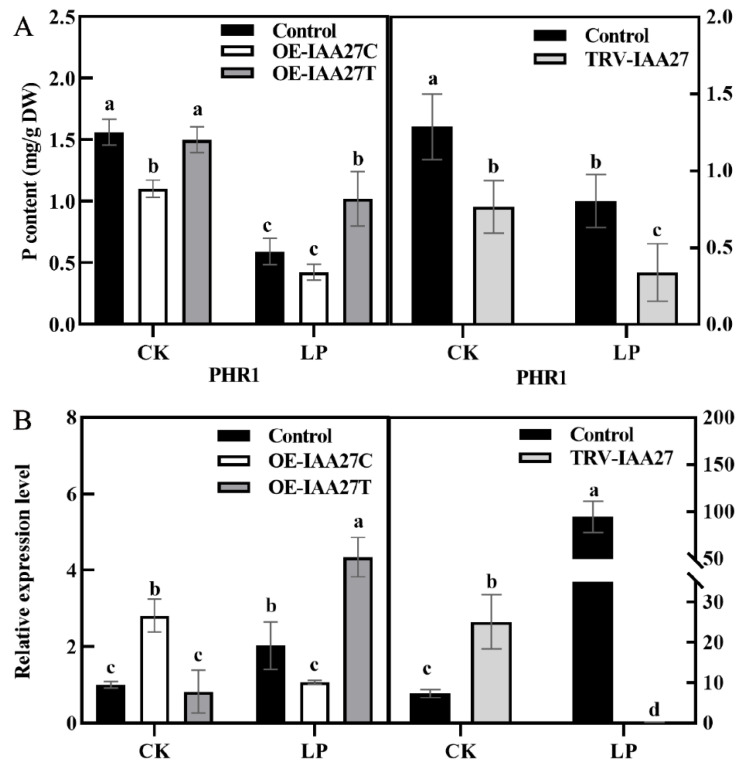
*IAA27* influenced adventitious root development and P uptake. (**A**) The P content of roots in transgenic and control apples after low phosphorus treatment. (**B**) *PHR1* expression was analyzed by qRT-PCR in *MdIAA27*-overexpressed and -silenced ‘M9’ plants. Error bars indicate standard deviations (s.d.) from three biological replicates. Different letters represent significant differences (*p* < 0.05).

The expression level of *MdIAA27* was induced by P deficiency stress, indicating that it may play an important role in low phosphorus conditions. To investigate *MdIAA27*’s function under phosphorus deficiency, a transient expression system was used to overexpress or silence *MdIAA27* in the roots of ‘M9’. The *MdIAA27*-overexpressed lines (OE-*IAA27T*, OE-*IAA27C*) and *MdIAA27*-silenced lines (TRV-*IAA27*) were obtained (Supplemental Figure S2). To further clarify the function of *MdIAA27* to cope with phosphorus deficiency conditions, the expression of the P uptake gene *PHOSPHATE STARVATIO RESPONSE 1* (*PHR1*) was analyzed in the rootstocks. Five days after phosphorus deficiency treatment, the expression of *MdPHR1* was upregulated, and the phosphorus content was increased in the roots of *IAA27T*-OE lines compared with control lines (Figure 4A,B). Conversely, the expression of *MdPHR1* was downregulated, and the phosphorus content of roots was decreased in *IAA27C*-OE and *IAA27*-TRV lines in contrast to control lines (Figure 4A,B).

**Figure 5:**
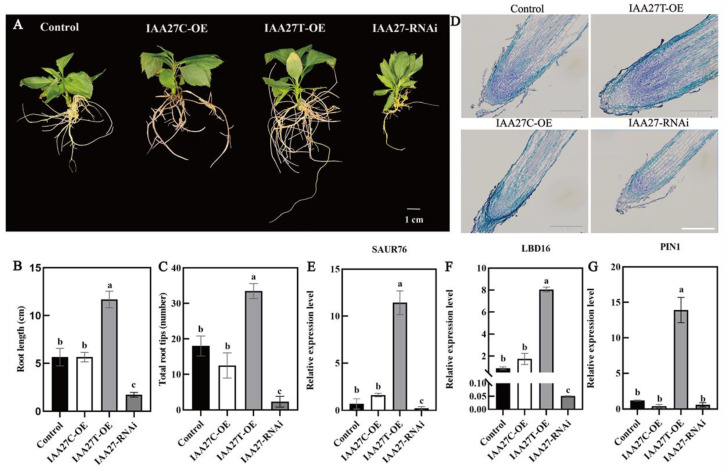
Overexpression and silenced lines of *IAA27* in ‘GL3’. (**A**–**C**) The adventitious root phenotypes of transgenic plants and control plants in the presence of auxin (0.5 mol/L) for 50 days on 1/2 MS solid medium; the experiment was repeated three times and the phenotype was consistent. (**D**) *MdIAA27* could influence the meristematic activity of root tips. Scale bars: 10 μm. (**E**–**G**) The gene expression of transgenic plants. Error bars indicate standard deviations (s.d.) from three biological replicates. Different letters represent significant differences (*p* < 0.05).

To further determine the role of *MdIAA27* in the phosphorus uptake of adventitious roots, *IAA27T*-OE, *IAA27C*-OE, and *IAA27*-RNAi were constructed and stably transformed into ‘Gala3’ (‘GL3’) apple plants, resulting in transgenic plants with significant increases or decreases in *IAA27* transcription (Supplemental Figure S3A–C). The empty vector was transformed as the control. Obviously, compared with the control, the numbers and lengths of the ARs were significantly higher when *IAA27T* was overexpressed, while no obvious change in root development in plants with the overexpression of *IAA27C* was observed. Following this, the *MdIAA27*-silenced plants exhibited a significant suppression of root development compared with the control plants (Figure 5A–C). To clarify whether the different root phenotypes of transgenic plants are due to changes in root cell division, adventitious root longitudinal sections for control and transgenic plants were obtained. The results showed that the meristematic activity of OE-*MdIAA27T* in the root tip was clearly higher than that in the control, while the meristematic activity of the other transgenic lines was significantly lower than that of the control (Figure 5D).

Auxin-related genes such as *SAUR76*, *LBD16*, and *PIN-FORMED 1 (PIN1)* play a significant role in modulating AR and LR formation. To verify whether *MdIAA27* regulates the expression levels of these genes, the transcript levels of *MdSAUR76*, *MdLBD16*, and *MdPIN1* were measured in *MdIAA27* transgenic apple lines and control plants. As shown in Figure 5E, *MdSAUR76*, whose homologs show a positive influence on meristematic activity in *Arabidopsis*, had higher expression in overexpressed *MdIAA27T* lines and had no influence on *IAA27C* lines compared to control plants. Furthermore, the expression levels of *MdLBD16* and *MdPIN1* were upregulated in *MdIAA27T*-OE lines but had no significant change in *MdIAA27C*-OE lines compared with controls (Figure 5F,G). These results showed that *IAA27T* positively regulated the expression of *MdSAUR76*, *MdLBD16*, and *MdPIN1*. However, the above auxin-related genes showed the opposite expression trend in *IAA27*-RNAi lines. Taken together, these results indicated that *MdIAA27T* activated the transcription of several auxin-related genes and positively modulated root development in apple.

**Figure 6:**
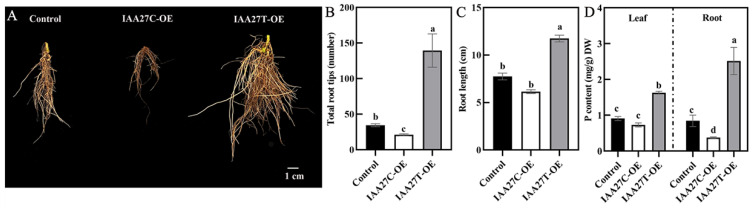
Effect of *MdIAA27* on phosphorus tolerance in transgenic apple plants. (**A**) The phenotypes and P content of 75-day-old transgenic and control apple plants treated with low phosphorus (LP, 10 μM KH_2_PO_4_) for 10 days. Transgenic plants were grown for 14 days with P and then transferred to a hydroponic solution lacking P for 10 days. (**B**,**C**) Statistical analysis of total root tips and root lengths. (**D**) Phosphorus content of transgenic and control lines after treatment with LP (10 μM KH_2_PO_4_) for 10 days. Error bars indicate standard deviations (s.d.) from three biological replicates. Different letters represent significant differences (*p* < 0.05).

These overexpression lines were further treated with low phosphate. After 10 days of growth in a solution containing 10 µM P, compared with the control plants, the *MdIAA27T*-OE transgenic apple plants revealed stronger adventitious root development, but the *MdIAA27C*-OE apples revealed attenuated growth in terms of adventitious root development (Figure 6A–C). Moreover, the P content both in the root and leaf tissue of *MdIAA27T*-OE plants was obviously the highest, but the lowest P content was observed in the whole plants of *MdIAA27C*-OE lines (Figure 6D). These results indicated that *MdIAA27* is a key regulator involved in apple for responding to P starvation.

### 2.4. IAA27 Acts as a Regulator of Root Development by Interacting with ARF

**Figure 7:**
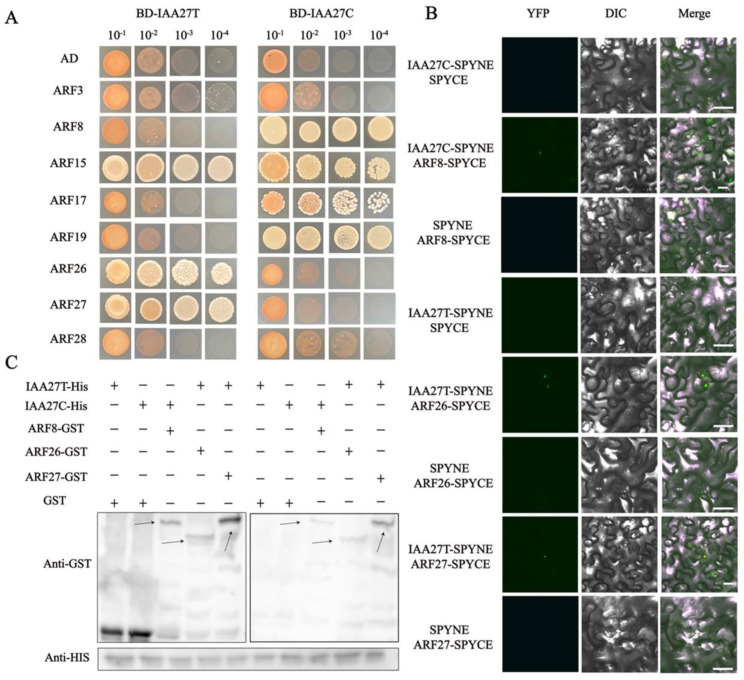
MdIAA27 interacts with MdARFs, which was identified by Y2H, BiFC, and pull−down assays. (**A**) Interaction between MdIAA27 and MdARFs in a Y2H assay. The ability of yeast cells to grow on synthetic medium lacking tryptophan, leucine, histidine, and adenine was scored as positive interaction. (**B**) Interaction between MdIAA27 and MdARFs as determined using a BiFC assay. MdIAA27−SPYNE and MdARFs−SPYCE were performed for the interaction assay. MdIAA27−SPYNE + SPYCE and SPYNE + MdARFs−SPYCE were used as negative controls. MdIAA27−SPYNE + MdARFs−SPYCE was used as a positive control. Scale bar = 10 μm. (**C**) Pull−down assay analysis of the MdIAA27−MdARFs interaction. The recombinant MdARF−GST and GST proteins were incubated with MdIAA27−HIS protein. The immunoblotting result was tested using anti−HIS and anti−GST antibodies. The plus and minus signs indicate the presence or absence of that protein, respectively. Black arrows indicate the positions of proteins. These assays were repeated three times with identical results.

In various plants, Aux/IAA proteins, as transcriptional regulators, mediate auxin signaling through protein–protein interactions with ARF members. The auxin response factors ARF3, ARF8, ARF15, ARF17, ARF19, ARF26, ARF27, and ARF28 were identified as potential MdIAA27-interacting proteins (Supplemental Table S2). Subsequently, Y2H assays were conducted to further validate the interactions between IAA27 and ARFs. The full length of *MdIAA27s* and *MdARFs* was inserted into the pGBKT7 (pGBD-*MdIAA27T* and pGBD-*MdIAA27C*) and pGADT7 (pGAD-*MdARFs*) vectors, respectively (Figure 7A,B). The results showed that MdIAA27-T interacted with MdARF15, MdARF26, and MdARF27, while MdIAA27-C interacted with MdARF8, MdARF15, MdARF17, and MdARF19.

To further verify the interactions between MdIAA27 and MdARF TFs, a pull-down assay was conducted in vitro (Figure 7C). The MdARFs-GST fusion protein and GST protein were separately incubated with the MdIAA27-HIS protein, and the MdIAA27-HIS protein was pulled down by MdARFs-GST, but not by the GST protein alone. A BiFC assay was carried out in *Nicotiana benthamiana* leaf cells to further investigate the MdIAA27-MdARFs interactions. The YFP signal was observed only when pSPYNE-MdIAA27 was co-expressed with pSPYCE-MdARF in the same cells, but was not detected in the other vector combination (Figure 7B). Collectively, these results showed that MdIAA27T physically interacted with MdARF26 and MdARF27, while MdIAA27C physically interacted with MdARF8.

### 2.5. SAUR76 and LBD16 Act Downstream of ARF TFs to Regulate AR Development

**Figure 8:**
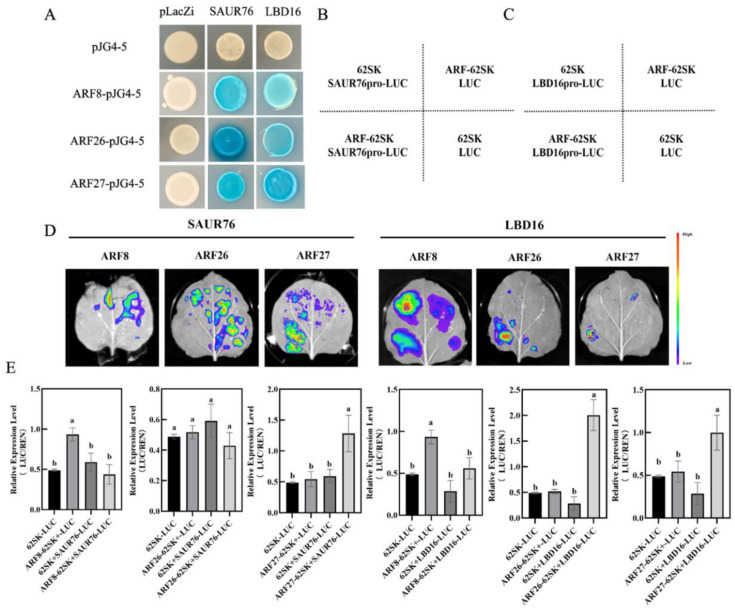
MdARFs directly bind to the promoter of *MdSAUR76* and *MdLBD16*. (**A**) Results of the Y1H, showing MdARF8, MdARF26, and MdARF27 binding to the *MdSAUR76* and *MdLBD16* promoter. (**B**–**E**) Effect of MdARFs on the regulation of the *MdSAUR76* and *MdLBD16* promoter in tobacco leaves and LUC/REN ratio analysis. Red color represents a stronger signal, and violet color represents a weaker signal. Error bars indicate standard deviations (s.d.) from three biological replicates. These assays were repeated three times with the same results. Different letters represent significant differences (*p* < 0.05).

To investigate the molecular mechanism by which *IAA27* influences the expression of *LBD16* and *SAUR76* genes, the 2000-bp upstream region in the promoter before the start translation codon ATG of *MdSAUR76* and *MdLBD16* was sequenced. There are three MdARFs-binding sites located at the *MdSAUR76* and *MdLBD16* promoters. Subsequently, yeast one-hybrid (Y1H) assays were conducted to confirm combinations between promoters of *MdSAUR76* and *MdLBD16* and all ARF genes in vitro. MdARFs bound to the *MdSAUR76* and *MdLBD16* promoter (Figure 8A).

Moreover, LUC/REN activity was also assessed using the *MdARF8*, *ARF26*, and *ARF27* cDNAs, which were cloned into the pGreenII62-SK vector to construct effector plasmids, separately. The 1984-bp *MdLBD16* and1968-bp *MdSAUR76* promoter were cloned into the pGreenII0800-LUC vector to generate the reporter construct. The effector and reporter constructs were introduced into tobacco leaf cells and transformation with the empty reporter plasmid was used as a control. In the LUC assay, MdARF27 significantly activated *MdSAUR76* and *MdLBD16* expression. MdARF26 clearly activated *MdLBD16* expression, but it did not show a significant change in *MdSAUR76* expression. By contrast, MdARF8 suppressed *MdSAUR76* and *MdLBD16* expression (Figure 8B–E). These results showed that ARF27 may play a more positive role in adventitious root development compared with other ARF members.

**Figure 9:**
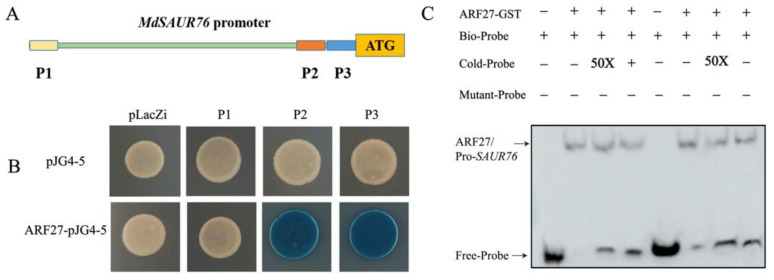
Interaction of MdIAA27 with the promoter of *MdSAUR76*. (**A**) Schematic diagram of the *MdSAUR76* promoter region; (**B**) Y1H confirmation of the binding of *MdSAUR76* promoter P2 and P3 fragments by MdARF27; (**C**) EMSA assay showing that MdARF27 could directly bind to the promoters of *MdSAUR76* (P2 and P3). The plus and minus signs indicate the presence or absence of that protein, respectively.

To further investigate the mechanism of *ARF27* and *SAUR76* regulating the root development in low phosphorus conditions, the promoter fragment was further truncated into three shorter sequences with ARF binding sites, named P1, P2, and P3, respectively (Figure 9A). Y1H assays indicated that these combinations were also observed with both fragments P2 and P3 (Figure 9B). In addition, the electrophoretic mobility shift assay (EMSA) affirmed that ARF27-GST bound to the P2 and P3 segment in the promoter of *MdSAUR76* (Figure 9C).

## 3. Discussion

### 3.1. MdIAA27 Responds to Phosphorus Deficiency Stress

Numerous strategies to cope with nutrient stress have been suggested, among which transcription factors involved in responsive mechanisms have great potential. The *Aux/IAA* family is normally involved in responding with biotic and abiotic stress [37]. In previous studies, *MdIAA24* was reported to enhance cadmium tolerance by reducing Cd accumulation [38]; *MdIAA9* was observed to confer osmotic tolerance [39]; *OsIAA6* positively regulated drought resistance [40]. However, reports on the function of Aux/IAA TFs in alleviating phosphorus stress in apple are still rare. Therefore, based on the BSA results of a previous study, we identified and cloned a candidate gene, *MdIAA27*, from ‘BC’ and ‘M9’ and further investigated the molecular basis underlying the regulation of phosphorus deficiency tolerance in apple. Aux/IAA protein domains are highly conserved and are normally localized in the nucleus, such as SlIAA9 and GmIAA27 [35,36]. The subcellular location indicated that MdIAA27 was located in the nucleus (Figure 2B). Hence, MdIAA27 might act as a transcriptional regulator to affect the expression of downstream genes in the nucleus. The expression pattern of *MdIAA27* exposed to phosphorus deficiency showed that *MdIAA27* was significantly induced by 10 µM KH_2_PO_4_ and markedly upregulated by KH_2_PO_4_ treatment for 5 days. In different tissues, the expression patterns of *MdIAA27* were differentially induced. In adventitious roots, the expression of *MdIAA27* was induced the most (Figure 2A). Taken together, these results suggested that MdIAA27, a transcription factor similar to other Aux/IAA members, was involved in apple’s response to phosphorus stress. The P content determination of *MdIAA27* transgenic apple plants after low phosphorus treatment also supported this conclusion (Figure 4A,B). The stable overexpression of *MdIAA27T* in apple and tobacco showed more tolerance to low phosphorus, as determined by the identification of longer and denser roots, higher P content levels, and better growth outcomes (Figure 3 and Figure 6). These results further demonstrated that *MdIAA27* is highly associated with resistance to phosphorus stress in apple.

### 3.2. MdIAA27 Regulates the Number and Length of Adventitious Roots to Enhance Phosphorus Uptake

Several studies have shown that Aux/IAA TFs are also involved in root growth and development. For instance, *SlIAA15*-suppressed plants exhibit increased lateral root formation [41]. Recently, gain-of-function mutants of the *OsIAA13* gene in rice also displayed defects in the growth of lateral roots, which are closely associated with the transcriptional regulation of the set of genes participating in lateral root initiation [42]. However, few studies have revealed the functions of *Aux/IAA* in woody plants, especially in apple. In this study, the phenotypes of progenies showed that the adventitious root lengths and number of the *MdIAA27T* genotype were greatly higher than those of the *MdIAA27C* genotype after low phosphorus treatment (Figure 1D). Overexpressing *MdIAA27C* resulted in fewer ARs than the control lines (Figure 5A), which was similar to the overexpression of *AtIAA8* [43]. Interestingly, in our study, the number of adventitious roots and root length were greatly higher in *IAA27T*-OE lines compared with the control plants (Figure 5A). A similar phenotype was observed in *OsIAA4* transgenic lines [44]. The phenotype of RNAi-*IAA27* lines exhibited significant defects in adventitious root length and number compared to control plants (Figure 5A). These results indicated that *MdIAA27* is a critical regulator of adventitious root development. Adventitious roots commonly increase the absorption area of roots and enhance their abilities to assimilate nutrients and support plants [45]. Previous studies have shown that adventitious roots promote phosphorus absorption under phosphorus deficiency conditions. In rice, overexpression of *OsMYB2* stimulated adventitious root growth and, subsequently, phosphorus absorption in shoot and roots [22]. In our study, it was also observed that *MdIAA27T* overexpression lines increased the phosphorus content after low phosphorus treatment (Figure 3D and Figure 6D). The higher phosphorus content of *IAA27T*-OE plants is due to the enlarged growth of adventitious roots by *MdIAA27T*, which in turn causes an increased surface area, leading to improved phosphorus uptake in apple.

Under phosphorus deficiency, root length significantly increased for better P uptake. The activity of meristematic cells in the root meristem influences root elongation [46]. In previous research, overexpression of *AtIAA1* in *Arabidopsis* exhibited a significantly suppressed cell length and number [47]. In rice, the mutant of *GLU3* was observed, which had a defect in root cell division to inhibit root development [48]. In our study, differences in root meristematic activity were also observed in these transgenic apple plants. *AtSAUR76* was found to positively control root development by influencing meristematic activity and cell elongation [49]. As shown in Figure 5A–E, the long roots of overexpression *MdIAA27T* lines were due to the increased expression of *MdSAUR76* regulated by *MdIAA27T*, which caused improved meristematic activity, leading to a longer root phenotype. Similarly, the short roots of *IAA27*-RNAi plants might be due to the suppression of *MdSAUR76* expression, which in turn caused the arrest of cell division, resulting in more a severe root development defect phenotype. In addition, the overexpression of *MdIAA27T* and *MdIAA27C* in tobacco plants significantly influenced their root length (Figure 3A,B). Collectively, this observation proved that the root length was increased after promoting the root meristematic activity of apple, leading to the improvement in phosphorus uptake in roots.

Previous studies have indicated that auxin-inducible genes *PIN1* and *LBD16* are required for root development in plants [50,51]. In apple, overexpression of *MdPIN1* led to the accumulation of auxin and increased the adventitious root development [52]. Overexpression of *AtASL18*/*LBD16* induced root formation in *Arabidopsis* [53]. In this study, silenced apple plants led to lower *PIN1* and *LBD16* expression, while the expression of *PIN1* and *LBD16* was significantly higher when *IAA27T* was overexpressed. These results clarified that *MdIAA27* directly or indirectly regulated some auxin-related genes, i.e., *PIN1* and *LBD16*, to affect adventitious roots development.

### 3.3. Low Phosphorus Regulates Adventitious Root Development through the Aux/IAA–ARF–SAUR76/LBD16 Signaling Pathway in Apple

During plant growth, auxin regulates root development through the Aux/IAA–ARF pathway. In *Arabidopsis*, AtIAA19 together with ARF7 regulated lateral root formation [54]. The ARFs act as regulators of root formation. The *AtARF17*-overexpressing plants produced fewer adventitious roots, suggesting that *ARF17* plays a negative role in the development of adventitious roots [55]. On the contrary, *ARF6* and *ARF8* act as positive regulators of adventitious root formation [56]. Consistent with previous studies, Y2H, BiFC, and pull-down experiments were performed to reveal that MdIAA27T could interact with the positive regulators, including MdARF26 and MdARF27, while MdIAA27C could interact with the negative regulators MdARF17 and MdARF19 and a positive regulator, MdARF8 (Figure 7). These results indicated that allele mutants (C to T) can result in differences in binding to ARF and, consequently, differences in auxin response genes’ expression levels. In addition, these findings indicate that the roles of the IAA27-ARF TF modules in adventitious root growth are similar to those of other known Aux/IAA–ARF modules.

In plants, ARF TFs directly activate *SAUR* and *LBD* genes, regulating lateral and adventitious root development. The *AtSAUR15* acts downstream of ARF TFs to promote LR and AR formation in *Arabidopsis* [57] and the *AtLBD16* and *AtLBD18* as target genes of ARF7 and ARF19 to control AR formation [58]. In our study, the expression of *MdSAUR76* and *MdLBD16* in *MdIAA27* transgenic plants was significantly upregulated with the overexpression of *MdIAA27T*, suggesting that *MdSAUR76* and *MdLBD16*, as downstream genes of MdIAA27, regulate AR development in response to phosphorus stress. Y1H, dual-luciferase expression, and EMSA assays were conducted to determine the specific binding site of MdARF TFs to the *MdSAUR76* and *MdLBD16* promoter. The results fully verified that MdARF26 and 27 activated the expression of *MdSAUR76* and *MdLBD16*, and MdARF8 weakly repressed the expression of *MdSAUR76* and *MdLBD16* (Figure 8 and Figure 9). In addition, it was observed that phosphate starvation response gene *MdPHR1* showed significantly enhanced expression levels in *MdIAA27T*-OE lines but decreased expression in the *MdIAA27C*-OE and *MdIAA27*-TRV lines after LP treatment. These results indicated that the allele mutant of MdIAA27 directly or indirectly influences the expression of *PHR1*, which potentially further affects the absorption of phosphorus, but the specific mechanism remains to be explored.

**Figure 10:**
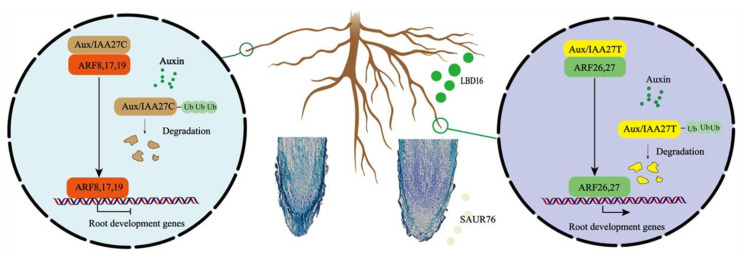
A working model of MdIAA27-regulated adventitious root growth and development in response to phosphorus stress. Yellow domains denote the IAA27-T mutant proteins and brown domains denote the IAA27-C proteins. Green domains denote the positive ARF proteins and red domains denote the negative ARF proteins. Arrows indicate positive effects, whereas lines ending with a short bar indicate suppressive interactions.

Our results together show a model for MdIAA27 to regulate the development of adventitious roots and increase tolerance to low phosphorus stress in apple rootstocks (Figure 10). The mutation (C to T) in the CDS of *IAA27* results in a changed interaction from negative regulators (ARF8, ARF17, and ARF19) to positive regulators (ARF26, ARF27). When low phosphorus stress occurs, it leads to the degradation of IAA27 and release of the ARFs. The degradation of IAA27T can release the positive ARFs, which further upregulates the expression of *SAUR76* and *LBD16* to improve the length and number of AR, while the degradation of IAA27C releases the negative ARFs to suppress the expression *SAUR76* and *LBD16* to inhibit the length and number of AR. Therefore, our studies provide new insights into the molecular crosstalk between adventitious root development and P starvation in apple plants.

## 4. Materials and Methods

### 4.1. Plant Growth and Treatments

Propagation of ‘BC’ × ‘M9’ F1 hybrids was performed by shoot cutting. After rooting, well-growing and healthy progenies were transferred into 1/2 strength Hoagland solution with 500 µM KH_2_PO_4_ for 14 days and then recorded by photographing.

To determine the root phenotype under low phosphorus conditions, extreme progenies were cultured in 8 L 1/2 strength Hoagland solution with 10 µM KH_2_PO_4_ (LP) for 30 days. To determine the expression levels of *IAA27* genes, ‘M9’ was cultured in 8 L 1/2 strength Hoagland solution with 500 µM KH_2_PO_4_ (CK) and with 10 µM KH_2_PO_4_ (LP), respectively. Roots, stems, and leaves were collected every five days and immediately placed in liquid nitrogen, and then stored at −80 °C.

‘GL3’ (*Malus domestica*) and tobacco (*N. benthamiana*) used for the transformation experiment were cultured on Murashige and Skoog solid medium containing 0.2 mg/L indole-3-butyric acid (IBA) and 0.6 mg/L 6-benzyl amino purine (6-BA) with a pH of 5.8–6.0 under a 16 h:8 h (light:dark) photoperiod at 22 °C. Tobacco seedlings were used for subcellular localization, BiFC, and dual-luciferase assays and these plants were grown at 22 °C under long-day conditions (16 h:8 h, light:dark). In order to evaluate the phenotype traits of roots and quantify P content, all transgenic plantlets were treated with 1/2 strength Hoagland solution, and then roots and leaves were harvested at 10 days after treatments.

### 4.2. Vector Construction and Genetic Transformation

The intact *MdIAA27* CDS was inserted into the PRI101 vector containing the 35S promoter to generate the *MdIAA27*-OE vector. For RNAi constructs, a 400-bp fragment was also inserted into the PRI101-RNAi vector.

Apple hairy root transformation: *MdIAA27T*-OE, *MdIAA27C*-OE, *MdIAA27*-RNAi vectors and empty vectors were transferred into *Agrobacterium rhizogenes* strain K599 and introduced into ‘GL3’ stems [59,60,61]. The *A. rhizogenes* positive colonies were transferred to LB liquid medium containing 50 mg/L rifampicin and kanamycin by shaking at 28 °C for approximately 10 h. The bacterial liquid was centrifuged for 8 min at 7000× *g* and resuspended with MS buffer (4.43 g/L MS, pH 5.6, 30 g/L sucrose, and 100 μM acetosyringone) to a final OD600 of 0.6–0.8.

Tobacco genetic transformation: *MdIAA27T*-OE and *MdIAA27C*-OE construct plasmids were transferred into *A. tumefaciens* strain GV3101 and introduced into tobacco by the Agrobacterium-mediated leaf disk method [62,63]. Transgenic lines were then selected on kanamycin (50 mg/L).

The transgenic apple and tobacco were analyzed by PCR to confirm their identity. Primers are listed in Supplementary Table S1.

### 4.3. Subcellular Localization

The coding sequence of *MdIAA27* was amplified and inserted into the PRI101-GFP expression vector. Vectors were transformed into tobacco leaf cells through PRI101-GFP as a control. Two days after infestation, GFP fluorescence was observed using confocal laser scanning microscopy, FV3000 (Olympus, Tokyo, Japan). Primers are listed in Supplementary Table S1.

### 4.4. Agrobacterium-Mediated Transient Transformation of Apple Plants and Treatments

For the overexpression of *IAA27*, the coding sequences of *IAA27*C and *IAA27*T were amplified by PCR using the ‘BC’ and ‘M9’ cDNAs as a template, and the fragment was inserted into the PRI101 vector. To silence *IAA27* expression using VIGS [64,65], a 400-bp CDS of *IAA27* was inserted into the TRV2 vector to construct the pTRV-*IAA27* plasmid. The recombinant vectors were introduced into *A. tumefaciens* strain GV3101, and the resulting positive *A. tumefaciens* lines containing the plasmids were introduced into ‘M9’ seedlings using vacuum infiltration. The ‘M9’ seedlings were infected under a vacuum (over 0.08 MPa); five seedlings per gene were used for each treatment. The vacuum was released slowly and thereafter plants were washed in deionized water to remove excess bacterium solution and kept in water for 3 days at 22 °C, which was followed by growing in a 1/2 strength Hoagland solution. After 5 days, the fresh roots were used for RNA isolation and qRT-PCR analysis. Primers are listed in Supplementary Table S1.

### 4.5. Pull-Down Assays

The *MdIAA27T* and *MdIAA27C* CDSs were inserted into the pGEX4T-1 vector; *MdARF8*, *ARF26* and *ARF27* cDNAs were inserted into pET32a. The *MdIAA27*-pGEX4T-1, empty pGEX4T-1 vectors, *MdARF8*-pET32a, *MdARF26*-pET32a, and *MdARF27*-pET32a were respectively transformed into BL21 (Weidi Biotechnology Co, Ltd., Shanghai, China). Transformed cells were treated with 0.5 mM isopropyl β-D-1-thiogalactopyranoside (IPTG) for 14 h at 16 °C. Pull-down assays were performed as described in [66]. Finally, the proteins were detected by a Western blot assay with anti-GST and anti-His antibodies (CoWin Biosciences, Beijing, China), as previously reported [65,66]. Primers are listed in Supplementary Table S1.

### 4.6. Bimolecular Fluorescence Complementation (BiFC) Assay

Full-length *MdIAA27T* and *MdIAA27C* CDSs were cloned into the 35S::pSPYNE vector (*MdIAA27T*-SPYNE, *MdIAA27C*-SPYNE) and *MdARF8*, *MdARF26*, and *MdARF27* were cloned into the 35S::pSPYCE vector (*MdARF8*-SPYCE, *MdARF26*-SPYCE, and *MdARF27*-SPYCE). These confirmed constructed vectors were used for transformation into GV3101 Agrobacterium separately. Various combinations of these vectors were co-expressed in tobacco leaves. The tobacco leaves were observed under a FV3000 confocal microscope (Olympus, Tokyo, Japan) to examine the fluorescence signal. Primers are listed in Supplementary Table S1.

### 4.7. Y1H and Y2H Assays

The *MdARF8*, *MdARF26*, and *MdARF27* CDSs were inserted into the pJG4-5 vector (Clontech, USA), and the *MdSAUR76* promoter (full-length, −2001 to −33 bp; P1 fragment, −2001 to −1850; P2 fragment, −467 to −341; P3 fragment, −341 to −258) and *MdLBD16* promoter were cloned into the pLacZi vector. The constructed fusion and empty vectors were introduced into yeast strain EGY48 (Weidi Biotechnology 86 Co, Ltd., Shanghai, China). The Y1H assay was conducted as previously described [67]. The yeast cells were grown on SD/-Leu/-Trp-deficient medium at 28 °C for 3 days.

For the Y2H assay, *MdIAA27T* and *MdIAA27C* CDSs were cloned into pGBKT7 and *MdARFs* CDSs were cloned into pGADT7 vectors. All constructs and empty vectors were introduced into the yeast strain Y2H (Weidi Biotechnology 86 Co, Ltd., China), as described in the Yeast Protocols Handbook (Clontech, Shanghai, China). Yeast cells were cultured on a minimal Leu and Trp medium for 3 days at 28 °C and then plated onto the SD (-Leu, -Trp, -His, and -ade) medium to analyze possible interactions. Primers are listed in Supplementary Table S1.

### 4.8. Dual-Luciferase Reporter Assay

For the LUC/REN activity assay, *MdARF8*, *MdARF26*, and *MdARF27* CDSs were cloned into the pGreenII62-SK vector to construct the effector; *MdSAUR76* and *MdLBD16* promoters were cloned into the pGreenII0800-LUC vector to construct the reporter plasmid. The effector and reporter constructs were introduced into Agrobacterium tumefaciens strain GV3101 (pSoup). After positive colonies were isolated, the bacterial liquid mixture of effector and reporter (9:1) was introduced into leaf cells by agroinfiltration. After 3 days, the LUC imaging was performed using a plant imaging system, LB985 NightSHADE (Berthold Technologies, Wildbad, Germany). The LUC/REN activities were detected and measured using the Dual-Luciferase^®^ Reporter Assay System (Solarbio, Beijing, China) on a Glomax 20/20 Luminometer (Promega, Madison, WI, USA). Primers are listed in Supplementary Table S1.

### 4.9. EMSA

The *MdARF27* CDS was amplified and inserted into the pGEX4T-1 vector. The resulting *ARF27*-GST fusion plasmids were transformed into the BL21 (DE3) strain of Escherichia coli. The expression of the ARF27-GST fusion protein was induced with 0.5 mM IPTG at 16 °C for 14 h. The recombinant protein ARF27-GST was purified using a GST-Tagged Protein Purification Kit (Soluble Protein) (CWBIO, Beijing, China). The *SAUR76* promoter fragments containing two ARF binding sites were synthesized as the Cold Probe and Bio-Probe. EMSA was performed using a Chemiluminescent EMSA kit (Beyotime, Shanghai, China). Primers are listed in Supplementary Table S1.

### 4.10. Determination of Phosphorus Content

Leaves and roots were harvested on the 10th day after low phosphorus treatment. The leaves and roots were dried for 3 days in an oven at 65 °C until a constant weight was reached, and the dry weight was recorded. Dried plants were pulverized in an electric grinder and then digested with HNO_3_ in a microwave oven (Mars, CEM, Matthews, NC, USA). The phosphorus content was measured via inductively coupled plasma optical emission spectrometry (ICP-OES, Optima 5300DV, PerkinElmer, Waltham, MA, USA).

### 4.11. Accession Numbers

The accession numbers for genes investigated in this study are as follows: IAA27 (MD17G1189100), ARF3 (MD16G1239300), ARF8 (MD17G1131500), ARF15 (MD03G1119500), ARF17 (MD15G1359400), ARF19 (MD00G1103900), ARF26 (MD04G1096900), ARF27 (MD15G1221400), LBD16 (MD05G1200900), SAUR76 (MD07G1220700).

## 5. Conclusions

Overexpression of *MdIAA27* affected the root morphologies by increasing the length and number of adventitious roots in response to low phosphorus stress. The allele variation of MdIAA27 directly interacted with ARF8, ARF26, and ARF27, which affected the expression of the downstream *MdSAUR76* and *MdLBD16* genes involved in adventitious root growth and development. The mechanisms behind the allele variation of *IAA27* involved in the apple plants’ root development in response to phosphorus stress were indicated and provide a basis for further investigation relating to root development regulated by the auxin response process under phosphorus stress.

## Data Availability

Not applicable.

## Supplementary Materials

The following supporting information can be downloaded at https://www.mdpi.com/article/10.3390/ijms232214029/s1.
