# Correction: Zhao et al. Transcription Factor IAA27 Positively Regulates P Uptake through Promoted Adventitious Root Development in Apple Plants. *Int. J. Mol. Sci.* 2022, *23*, 14029

**DOI:** 10.3390/ijms26146739

**Published:** 2025-07-14

**Authors:** Shuo Zhao, Xuewen Zhao, Xuefeng Xu, Zhenhai Han, Changpeng Qiu

**Affiliations:** 1 College of Horticulture, China Agricultural University, Beijing 100193, China; 2 Key Laboratory of Stress Physiology and Molecular Biology for Fruit Trees in Beijing Municipality, China Agricultural University, Beijing 100193, China

In the original publication [1], there was a mistake in Figures 1D, 8A and 9B, as published. The authors sincerely apologize for this oversight and confirm that these errors were caused by a software layout problem and unintentional uploading of representative images. 

The corrected “Figure 1, Figure 8 and Figure 9” appear below. The authors state that the scientific conclusions are unaffected. This correction was approved by the Academic Editor. The original publication has also been updated.

## Figures and Tables

**Figure 1:**
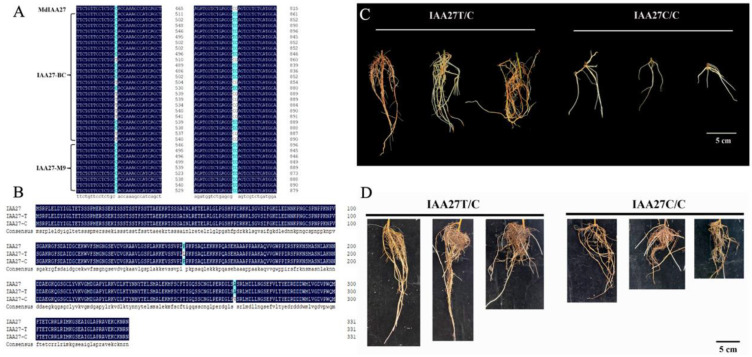
Identification of IAA27 related to root development in the parents ‘BC’ and ‘M9’ and their progenies. (**A**) Variation in the *IAA27* CDS sequence of the parents ‘BC’ and ‘M9’. (**B**) Amino acid sequence analysis of IAA27 transcription factors. (**C**) Phenotypes of adventitious roots from six progenies crossed from ‘BC’ and ‘M9’. The progenies were harvested for adventitious root trait analysis at 30 days after cutting. Scale bars: 5 cm. (**D**) After low phosphorus treatment for 30 days, adventitious roots of progenies were photographed. Scale bars: 5 cm.

**Figure 8:**
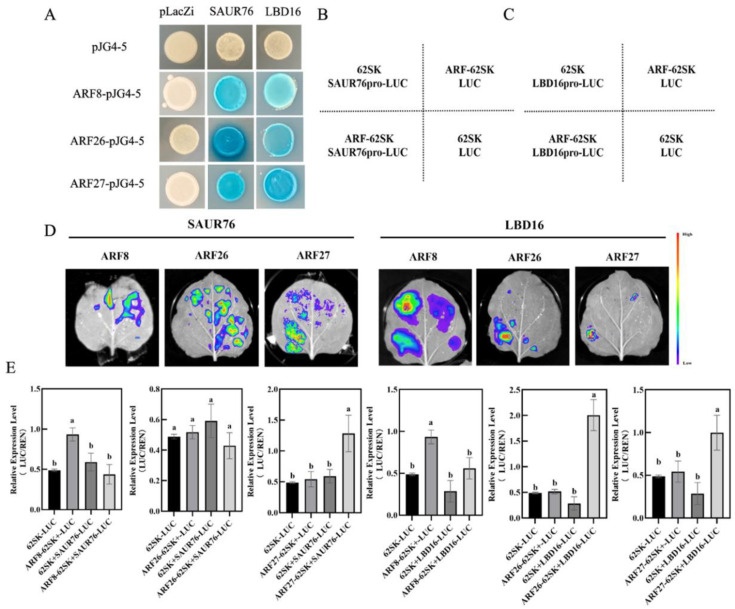
MdARFs directly bind to the promoter of *MdSAUR76* and *MdLBD16*. (**A**) Results of the Y1H, showing MdARF8, MdARF26, and MdARF27 binding to the *MdSAUR76* and *MdLBD16* promoter. (**B**–**E**) Effect of MdARFs on the regulation of the *MdSAUR76* and *MdLBD16* promoter in tobacco leaves and LUC/REN ratio analysis. Red color represents a stronger signal, and violet color represents a weaker signal. Error bars indicate standard deviations (s.d.) from three biological replicates. These assays were repeated three times with the same results. Different letters represent significant differences (*p* < 0.05).

**Figure 9:**
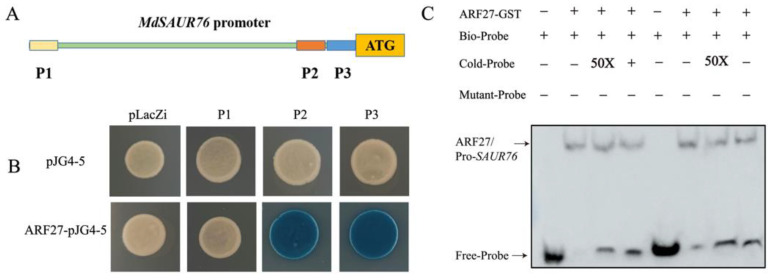
Interaction of MdIAA27 with the promoter of *MdSAUR76*. (**A**) Schematic diagram of the *MdSAUR76* promoter region; (**B**) Y1H confirmation of the binding of *MdSAUR76* promoter P2 and P3 fragments by MdARF27; (**C**) EMSA assay showing that MdARF27 could directly bind to the promoters of *MdSAUR76* (P2 and P3). The plus and minus signs indicate the presence or absence of that protein, respectively.
