# Supplementary material for: Transcription Factor IAA27 Positively Regulates P Uptake through Promoted Adventitious Root Development in Apple Plants
Source: Int J Mol Sci. 2022 Nov 14;23(22):14029. doi: 10.3390/ijms232214029 (PMC9695701; doi:10.3390/ijms232214029)
Supplement: Supplementary file 1 [file ijms-23-14029-s001.zip › Supplement Figure.pdf]

A

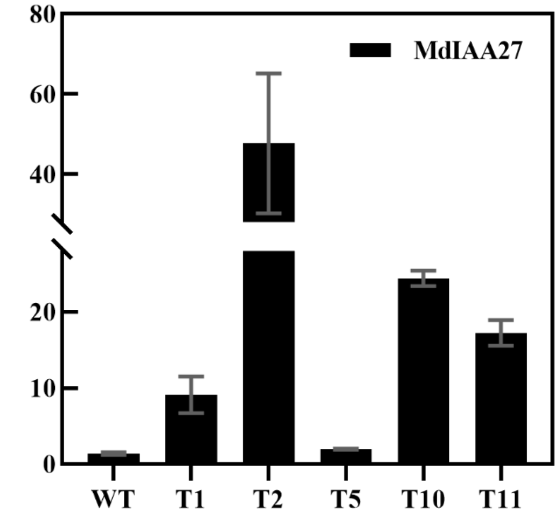
MdIAA27
Relative expression level
80
60
40
20
10
0
WT
T1
T2
T5
T10
T11

B

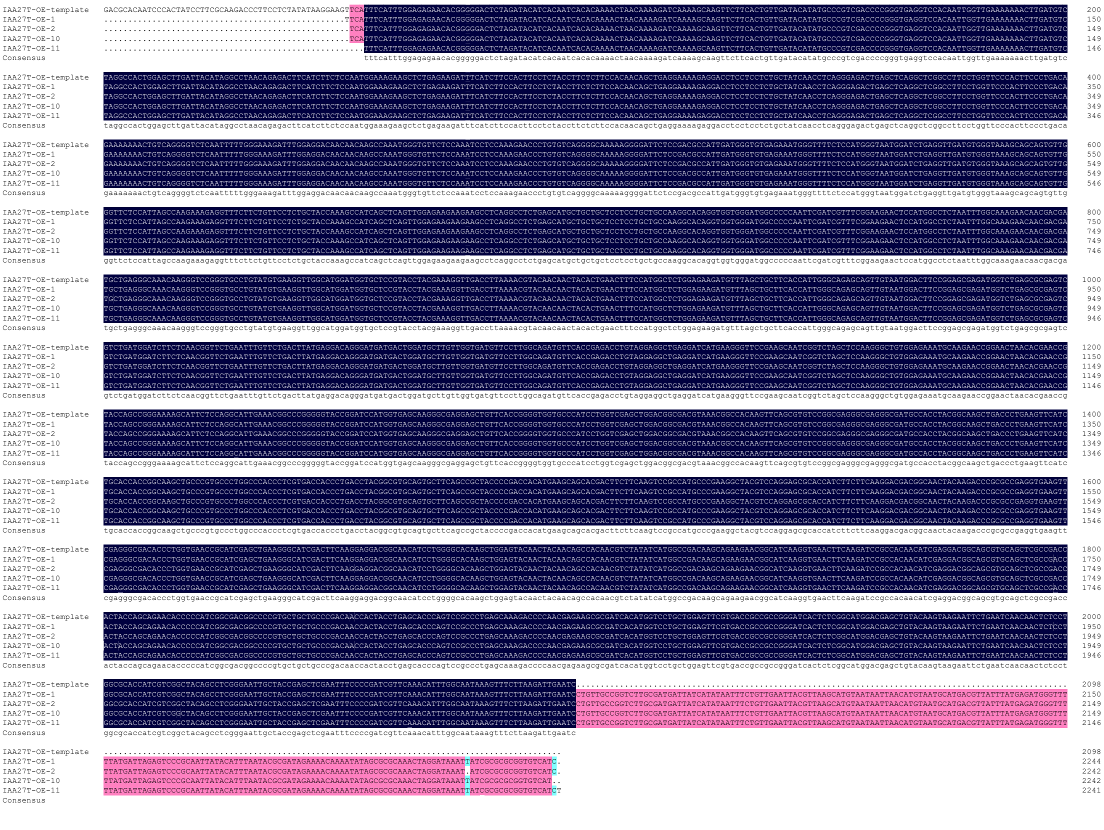
IAA27T-OE-template
IAA27T-OE-1
IAA27T-OE-2
IAA27T-OE-10
IAA27T-OE-11
Consensus

C

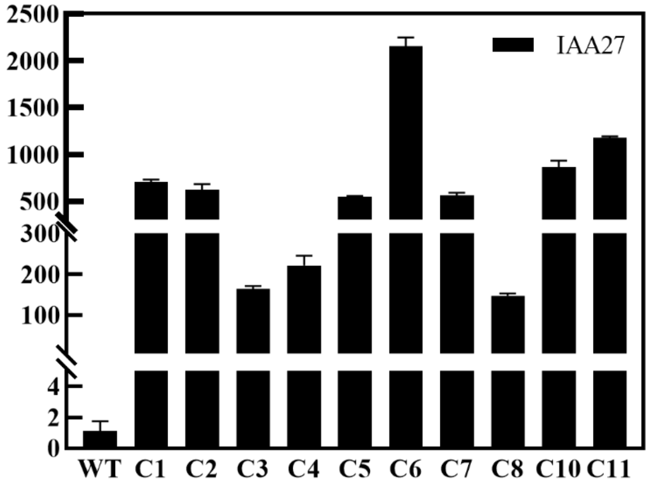

D

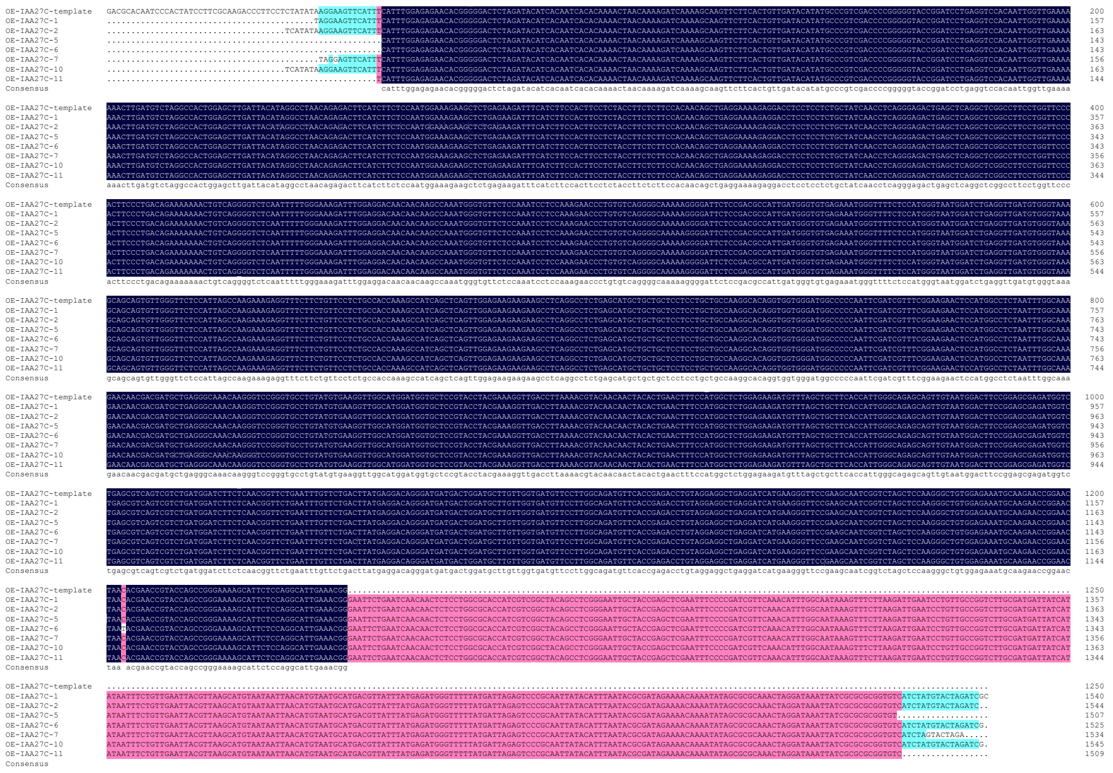

Figure. S1. Identification of *MdIAA27* transgenic plant in tobacco. (A, C) The relative expression of *MdIAA27* in transgenic lines was detected by qRT-PCR, *MxEF1α* was used as an internal reference gene. (B, D) Sequencing of PCR amplification products.

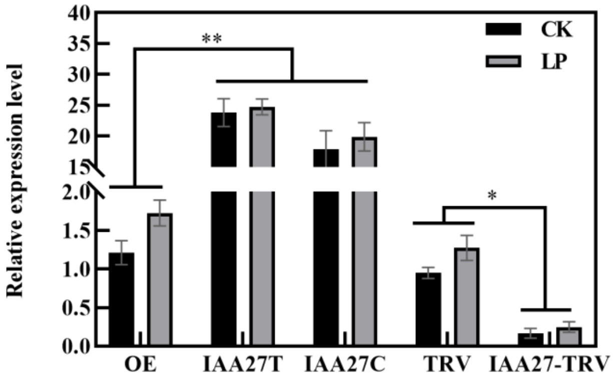

Figure. S2. Identification of *MdIAA27* transgenic plant in transient transformation apple. Transcript level of *MdIAA27* by qRT-PCR in control plants and transgenic apple plants (*IAA27T*-OE, *IAA27C*-OE and *IAA27*-TRV). * indicates statistically significant differences at $p \leq 0.05$. ** indicates statistically significant differences at $p \leq 0.01$. Error bars indicate standard deviation (s.d.) from three biological replicates.

A

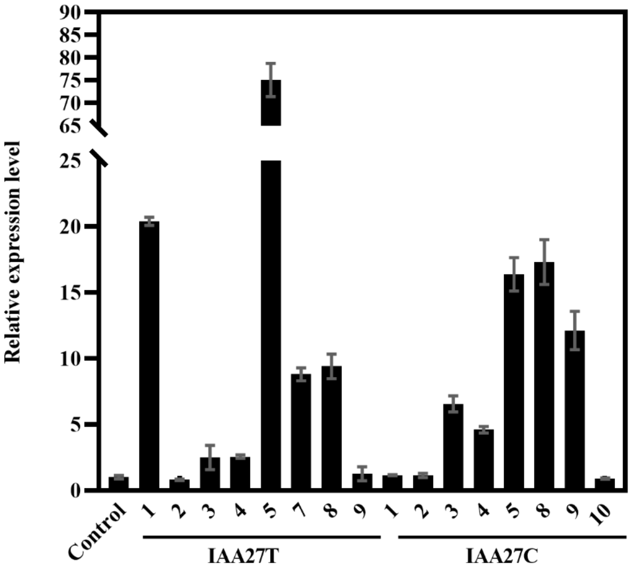

B

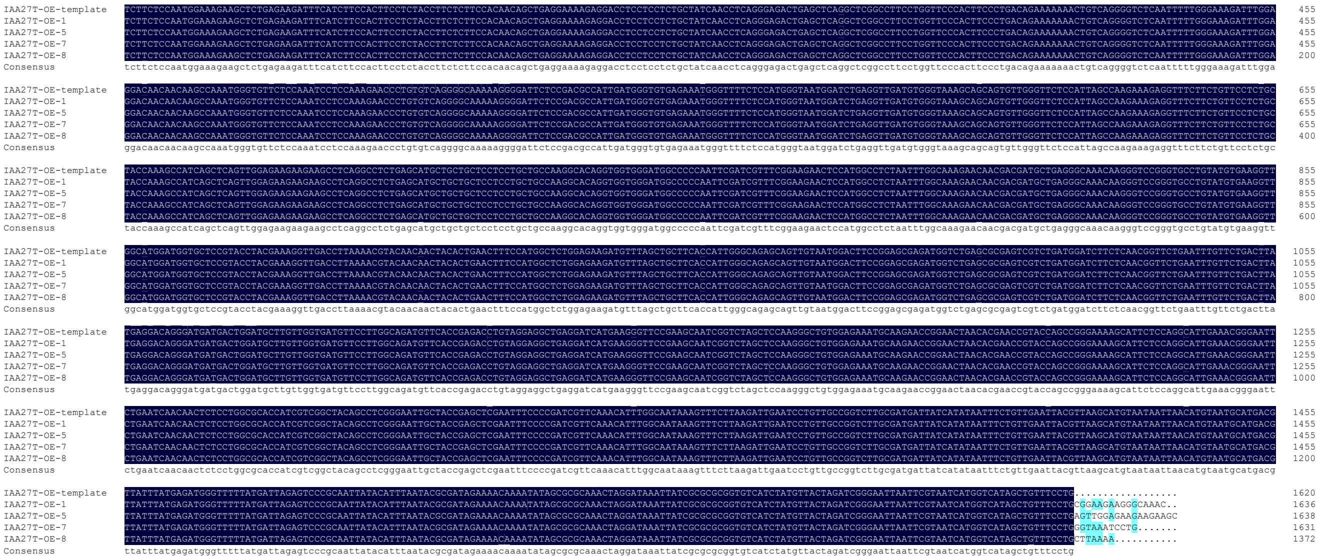

C

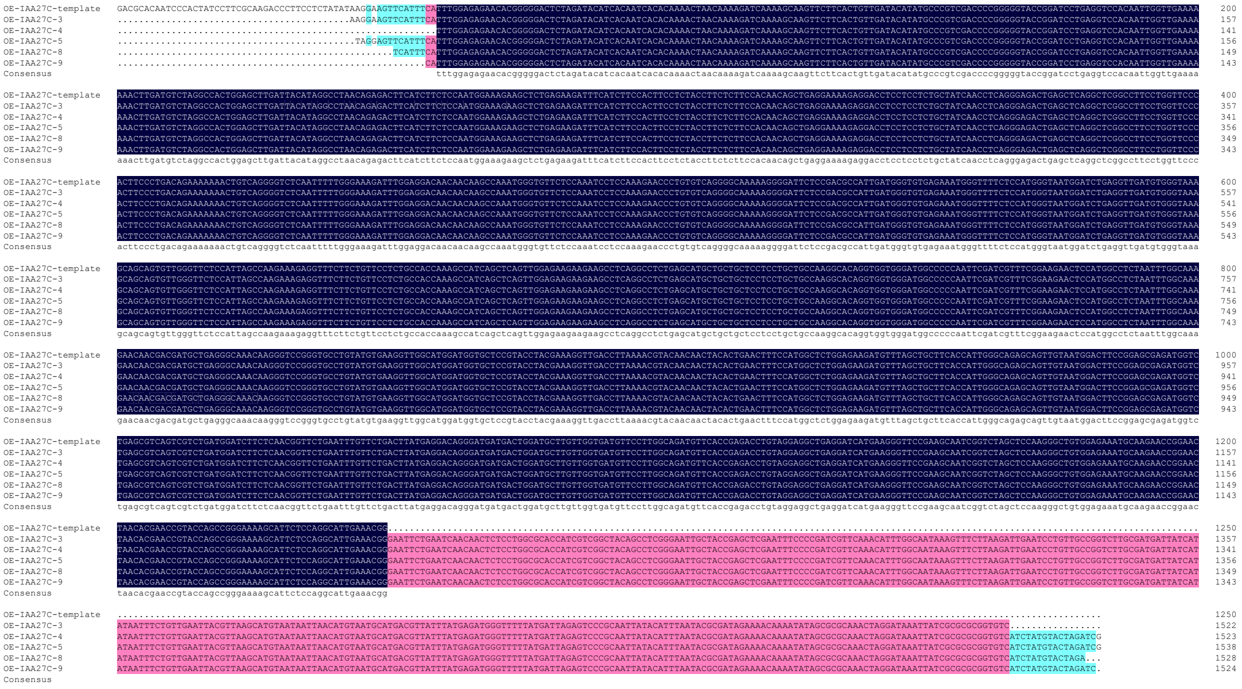

Figure. S3. Identification of *MdIAA27* transgenic plant in apple (A) The relative expression of *MdIAA27* in transgenic lines was detected by qRT-PCR, *MxEF1α* was used as an internal reference gene. (B-C) Sequencing of PCR amplification products.
